# The Hospital. Nursing Mirror

**Published:** 1898-04-30

**Authors:** 


"The Hospital" Nursing Mirror.

Being the Nursing Section of "The Hospital."

[Contributions for this Section of "The Hospital" should be addressed to the Editor, The Hospital, 28 & 29, Southampton Street, Strand, London, W.C., and should have the word "Nursing" plainly written in left-hand top corner of the envelope.]

### News from the Nursing World.

### THE QUEEN'S PARTING GIFTS TO NICE.

The Queen has sent £120 to M. Sauvan, the Mayor of Nice, to be distributed amongst the various benevolent institutions. Her Majesty has also sent the English Consul, Sir James Harris, a gift to be divided amongst the English charities.

### THE DUCHESS OF YORK AT PORTSMOUTH.

On the 25th inst., whilst the Duke of York inspected the cruiser, *Crescent*, the Duchess, accompanied by Admiral Sir Michael Culme-Seymour, visited the Sailors' Rest, and was conducted all over it by Miss Weston, the foundress. Her Royal Highness tasted the coffee at the bar, inspected the needlework of the sailors' wives, who have made garments for two of her babies, gave a further order, and finally wrote the following comment in the visitors' book, "Very much interested in all that I have seen."

### OPENING THE NURSES' INSTITUTE AT WEDNESBURY.

Civic festivities on a large scale marked the opening of the new nurses' institute at Wednesbury. At the Mayor's invitation the town was decorated and the day was observed as a general holiday. The Countess of Dartmouth, who was to perform the ceremony, and a large representative assembly were received at the Town Hall by the Mayor (Alderman J. Oldbury) and the Mayoress, and shortly afterwards a procession was formed, which passed along the Holyhead Road, Lower High Street, the Market Place, and Walsall Road to the new building. Here the Mayor introduced the Countess, after which Mr. Joynson, the architect, presented her with a golden key. Unlike many tokens of similar events it actually unlocked the door, and the visitors trooped in. Two bouquets were presented to the Countess on behalf of the ladies' committee, one by the Mayoress and one by the Ex-Mayoress (Miss Knowles) who also bestowed a like gift upon the Mayoress. After the usual proceedings luncheon was served at the Town Hall, at which one hundred and fifty guests were present. In the evening the Mayor and Mayoress gave a reception and ball. The new home, the building and plenishing of which has cost £1,426, contains ten rooms, and affords accommodation for two, or perhaps three, nurses. As there was a balance of £700 from the sum collected for the Jubilee memorial it has been invested as the nucleus of a maintenance fund. Gifts and subscriptions towards this fund amounting to £105 were promised during the day.

### READY FOR ACTION.

Rumours of war have brought nurses from their hives as the sun brings out the bee, and the declaration of war between Spain and America found the nursing service in the latter country fully equipped and ready for action. The American Government has fitted up the steamer *Creole* as a hospital ship, and the services of Miss Ella Enright, vice-president of the Graduated Nurses Protective Association, with a band of volunteer nurses, have been accepted for duty on board. A distinctive uniform has been designed. The dress is of navy blue India silk, made simply, but picturesquely. The bodice folds across in front, and the deep loose soft collar which is attached has the appearance of the folded neckerchief of puritan times. India silk was chosen, as this material is light and cool, and requires no ironing after washing. A large white bibless apron and a mob cap complete the indoor costume. Long cloaks and Alpine hats of navy blue are added for travelling. The American Red Cross Society's flag flies over the steamship *Texas*, which sailed from Key West for Cuba on the 23rd inst. Miss Rotty, a wealthy English lady, who is making the care of the sick and wounded her special work, is on board, besides a number of medical men and nurses. The *Texas* also carries over a thousand tons of supplies for the reconcentrados, and is under the protection of a man-of-war. Other English ladies are also offering their services as nurses at the American Embassy in London.

### POT-LUCK AT THE VICTORIA COMMEMORATION CLUB.

How many nurses rushing through the fatigues of business or pleasure know of the delightful resort provided for them in the Victoria Commemoration Club in Southampton Street, Strand? Those who do, value the elegant rooms, dainty service, and well-cooked repasts; they also appreciate the reasonable charges which rule there; and those who do not should lose no time in becoming acquainted with the place. At one p.m. the house lunch is served daily, and members may partake thereof for the small sum of 1s. The following is the meal provided on the 23rd inst.: Cold lamb, mint sauce, new potatos, and broccoli sprouts, followed either by a sweet—in this case rhubarb tart—or cheese, butter, and biscuits. Some little time ago, on a bitterly cold day, the guests found a delicious Irish stew, cooked to perfection, awaiting them, with jam tarts and lemon pudding to follow. The small cost of these excellent meals is owing to the fact that the club is a co-operation of nurses, and it is not intended to make more money out of it than will pay expenses.

### INDIAN PLAGUE NURSES.

There is a report abroad that great discontent prevails amongst the nurses engaged in the plague hospitals in India. It is said that, in consequence of the hospitals not being prepared with accommodation for their reception, the whole of each nurse's salary, excepting the small amount of 6s. per month, has been absorbed in rent and maintenance. We venture, however, to doubt whether this refers to the nurses sent out from England. Their agreement was that they should be provided with free quarters by Government; and from Poona, at any rate, we hear that they can live on a half to two-thirds of their salaries. Moreover, so far, no complaints have been made on this score at headquarters, and from what we have heard from those who have recently returned from India we have not gathered any suggestion of such dissatisfaction being prevalent. We hope to give an explanation at an early date.

### A WISE REGULATION.

Arrangements have been made for the nursing staff at the Seamen's Hospital, Greenwich, to have six months in a London hospital for women included in their three years' training.

### CHARITY EXTRAORDINARY.

Mr. Alfred Marriott, of Hopton Grange, Mirfield, Yorkshire, has bequeathed the enormous sum of £597,000 to charity. The largest legatee is the Society for the Propagation of the Gospel in Foreign Parts, which receives the handsome sum of £180,000. Churches, hospitals, and philanthropic societies in the dioceses of Canterbury, York, and of London and Rochester fare well, and institutions for women and children especially so. We quote a few in London that have benefited: London Branch of the Church of England Temperance Society and Police Court Mission, £2,000; London Lock Hospital and Rescue Home, £1,000; Home for Fallen Women, £2,000; Westminster Female Refuge for Fallen Women, £2,000; London Diocesan Penitentiary, £2,000; Society for Rescue of Young Women and Children, £1,000; Queen Charlotte's Lying-in Hospital, £1,000; National Refuges for Homeless and Destitute Children and the "Arethusa" and "Chichester" Training Ships, £2,000; North-Eastern Hospital for Children, £2,000; Alexandra Hospital for Children with Hip Diseases, £2,000; Church of England Incorporated Society for Homes for Waifs and Strays, £2,000; Boys' Home, Regent's Park, £2,000; East London Industrial School, Lewisham, £1,500; and Clergy Orphan Corporation, Parliament Street, £2,000.

### IN AID OF THE WESTMINSTER HOSPITAL.

A grand choral and orchestral concert is to be given at St. James's Hall on Wednesday evening, May 11th, at eight o'clock, by three associations; the Westminster Orchestral Society, and the Streatham and Reigate Choral Societies. The Princess of Wales and the Duke and Duchess of York have promised their patronage. Tickets may be obtained from Mr. Basil Tree's office, St. James's Hall, and Messrs. Hill and Sons, 140, New Bond Street, or from members of the concert management. Seats should be booked at once, as on and after May 1st the prices, which at present are very moderate, will be raised.

### A WOMAN INSPECTOR FOR THE POTTERIES.

A petition is about to be presented to the Home Secretary praying that the special inspector to be appointed for the Potteries may be a woman. The employés are chiefly women, there is no machinery, the district is a compact one, and the appointment seems to be eminently one which could best be filled by a lady, Amongst the signatures to the petition are those of the Duke and Duchess of Sutherland, Miss G. Tuckwell, president of the Women's Trade Union League, and of various representatives of trades unions in the district.

### NEW YORK NURSES' EDUCATIONAL EXHIBIT.

In conjunction with the International Health Exposition, now being held in the Industrial Building, Forty-third Street and Lexington Avenue, New York City, there is a Trained Nurses' Educational Exhibit. It remains open from the 25th inst. to May 31st.

### LECTURES ON PUBLIC HEALTH.

Miss Alice Ravenhill, late secretary to the Royal British Nurses' Association, has just been elected an associate of the Sanitary Institute. Miss Ravenhill has recently completed a very successful lecturing tour, extending over five months, undertaken under the auspices of the Women's Co-operative Guild, the subject of the lectures being "The Laws Framed to Protect and Promote Public Health."

### NURSING CANDIDATES.

It is reported that the enormous number of 5,000 candidates applied for nurses' training at one of the large London hospitals during the year. In spite of this statement, which is possibly true, for applicants are plentiful, it is well known that there is a dearth of suitable women, and a growing demand for them. The physical sifting is very severe, and in the best-managed institutions the medical fiat is issued before the health of the probationer is seriously affected. Would-be nurses who are not exceptionally strong should, therefore, choose a country hospital in preference to the town. Age, capacity, moral character are factors also in thinning the roll. Many folk fondly imagine that anyone can be a nurse if she were but trained. This, however, is a mistake. Training is essential, but training is not everything.

### SHORT ITEMS.

Mr. Britton's tender to build the new Jubilee Nursing Institute at Barry has been accepted by the executive committee. Mr. Britton is a local architect, and his estimate of the cost is £1,451.—Two European ladies have volunteered as resident nurses at the Rangoon Leper Asylum. There are 34 patients in this institution, and the committee have gratefully accepted the proffered services.—Lady Laura Riddings was re-elected president at the annual meeting of the Derbyshire Society for Providing Trained Nurses, and a scheme affiliating the association with that of Nottinghamshire was adopted.—The Galashiels branch of Queen Victoria's Jubilee Institute for Nurses has lately presented its eighth annual report to its supporters. In spite of many liberal gifts, and the approval which Nurse White wins alike from her association, superintendent, and patients, the income was £40 less than the expenditure, but probably this was due to extraordinary items, amongst which may be reckoned a Jubilee offering to the parent society of £25.—Through the kindness of the Hon. Mrs. Vernon Auchans, twelve patients of the Kilmarnock District Nursing Association were received into the Dundonald Home. The Ochiltree Home kindly admitted five of the society's patients.—Women have been recognised as eligible assistants to the physicians of the gynæcological wards of the Glasgow Royal Infirmary, and women students now enjoy the same facilities at the dispensary as those offered to men.—During 1897 several nurses have left the Glasgow Royal Infirmary to take up important posts which have been offered them in Brazil and Natal. The managers, whilst regretting their loss, cannot but feel gratified at this proof of the esteem in which the training given at the institution is held.

### Lectures to Surgical Nurses.

By H. A. LATIMER, M.D. (Dunelm), M.R.C.S. of Eng., L.S.A. of London, Consulting Surgeon, Swansea Hospital; President of the Swansea Medical Society; Lecturer and Examiner of the St. John Ambulance Association, &c.

### XXX.—AFTER-TREATMENT IN CASES INVOLVING ABDOMINAL OPERATIONS—PREPARATION OF THE PATIENT FOR OPERATION—ADMINISTRATION OF ENEMAS.

THE vomiting following the performance of ovariotomy is best treated by being left alone, or by the administration of a tea-spoonful of hot water at long intervals, or even by small bits of ice being swallowed. Dr. Whitla, in his admirable "Dictionary of Treatment," gives a word of caution about ice which, I think, will bear quotation. "The writer," he says, "has seen much mischief produced by ice administered too often, and in too great quantities. When thirst is great, Smith's plan of giving an enema of one pint of tepid water is often most efficacious. The fear of inducing vomiting should lead the nurse to give as little of anything as possible by the mouth. Water may accumulate in the stomach after ice has been given too freely, and vomiting may be thus produced." I have often known small quantities of tea, when given in these cases, act wonderfully well in checking sickness. The treatment of any complications which may arise after ovariotomy I may well leave here unnoticed in this lecture, for you will receive directions about such, should they occur, from the surgeons who are in attendance.

During these lectures on surgical nursing I have from time to time mentioned the fact of operations being performed, and I propose to-night to instruct you in your duties to the patients who are to undergo them. So much importance attaches to the efficient performance of these duties by you that your half of the work may be fairly said to almost equal in value that done by the surgeon, for however well the latter may have planned and executed his operation, his work may be entirely marred by your neglect in carrying out his instructions, and by your incompetency. It behoves you, therefore, to be thorough in all you do, and to be careful in attention to small details. If you watch the work of a successful surgeon you will see that the good results he obtains are due entirely to the care with which he plans his operations, and to the minute exactness with which he does his work. You must emulate him in the wards, taking care to be excessively clean in all you do, and never to be slovenly in the application of dressings and in the other duties entrusted to you.

An operation having been decided upon by the medical attendant, you will be told that the patient must be prepared by you to undergo it on a certain day and at a fixed hour, and you will now have to see that, in as far as lies in your power, he shall be brought into a state of mental and bodily fitness for the ordeal he is to undergo. The boldest man will naturally have some misgivings and timidity on such an occasion as this, and if he is left to brood over his trouble he is likely to be depressed and lowered in vitality by his gloomy thoughts and apprehensions. Try, then, to pass the time for him by giving him some suitable employment if he is capable of using his hands; and at all times assume a confident and encouraging air when talking to him about his illness, and what is about to be done to cure it. The influence of the mind over the body is wonderful, the general health being improved or lowered in direct response to a happy or gloomy train of thoughts. I cannot bear to hear an invalid constantly assert that he is not going to recover from an illness, for I have known many such a person be true to his statement, and die, apparently, from want of pluck. When epidemics of disease are prevailing, it is notorious that many people die of sheer depression; if they are affected at all with disease they may have it in so light a form that they should recover from it; but they resign themselves to their fate and succumb to their illnesses. And the reverse obtains to a notorious extent, many people having so much courage that they are sustained through pain and disease, and, having a confident belief in their power of recovery, get well when those around them can hardly hope for a successful issue to their cases. And I call you to notice one thing especially as proving what I have to say on this point. Observe how a belief on the patient's part in the capacity and power of his doctor helps him to bear his illness and to undergo operations performed upon him. You will see this constantly exhibited by hospital patients who will often willingly submit to them at the hands of one surgeon, whilst they shrink from allowing another to handle them in any way. Do all you can, at all times, to strengthen the authority of the medical man in attendance, with the view, not only of acting loyally towards him, but also of influencing for good the person who is under his charge.

So much for the mental preparations of the invalid; now for the bodily ones:—

It is essential that the whole of the skin of the patient shall be clean, and that the part which is to be operated upon shall have been rendered free from germs of disease. To effect these results, he must be washed well on the day before he is to undergo surgical treatment, and, on the day this is to take place, any parts which are to be dealt with are to be freely bathed with an antiseptic lotion, and shaved if they are covered with hair. After having been so dealt with, it is well to apply a moist antiseptic dressing to them, and to cover this with a waterproof protection, which is to be secured by bandaging. The bowels must have been relieved by an aperient given on the evening preceding the day of operation, and, if this aperient fails to act on the morning of this day, an enema of warm water is to be given to ensure a satisfactory evacuation taking place. Nothing is better for this purpose than simple warm water, of which at least one pint and a half should be quietly injected into the rectum. Enemas act by setting up reflex irritations; if they are sufficiently bulky they distend the bowel and provoke it to contract to expel its contents. It is important to remember this fact, and to know how to administer them in such a way that they shall not fail to secure the object for which they have been employed. Proceed as follows: After placing the patient on his left side, warm and oil the portion of the enema apparatus which is to be introduced into the bowel, and then gently place it in position; now cause the warm water to enter the gut, either by slowly repeated contractions of the hand on the ball of the syringe, or by a languidly-flowing stream from a douch, should you be using that form of instrument; after awhile you will be told by your patient that the bowels are about to act; now you are not to remove the nozzle, but simply stop working the instrument for a few minutes, recommencing when this feeling has passed off. After you have done this two or three times, the desire for an action of the bowels will be imperative, and complete relief will be afforded. If you are hasty in your pumping, or if you allow the patient to gratify his feeling of desire at the first call, the bowel will act inefficiently, and will not expel all its contents. Many a time, when operating upon rectal cases—for piles, or for fistula in ano—have I wished that enemas had been properly given. The patients have been placed on the table, and I have been assured that the bowels have acted properly after enemas, and yet it has been plain that this procedure has been carelessly carried out, for I have hardly commenced dilating the sphincter when it has been made clear that only a partial action of the bowels has been secured beforehand. This is the sort of thing I am alluding to when I tell you that you should be attentive to details.

### Post-Graduate Clinics for Nurses.

By a Trained Nurse.

### PRACTICAL POINTS IN LUNG DISEASES.—II.

A very troublesome element in some lung cases is the fact that expectoration frequently interferes with sleep to such a serious degree that the patient is quite worn out by this addition to his sources of exhaustion. An admirable plan to adopt with patients whose cough becomes troublesome on getting into bed, or on lying down for the night, is to use a graduated bed-rest, and gradually and slowly to lower them to a horizontal or semi-recumbent position.

Sudden changes of posture are apt to induce serious coughing fits in sensitive and phthisical patients, and such coughing fits are not only exhausting in themselves, but result in a material interference with sleep and rest. In the morning the same plan of gradually raising the patient by means of the graduated bed-rest from a lying-down to a sitting position should be observed. By such a simple expedient, these long, painful, and exhausting coughing spells may be very materially lessened, and the patient's strength saved. I have found that an arm chair with the legs cut off forms an excellent type of bed-rest in bronchitic and phthisical cases where any attempt to lie down produces violent coughing. The patent sits in such a comfortable pillow-packed bed-rest, warmly covered and cosy, and soon learns to get a good night's rest in his "bed chair."

Coughing fits may sometimes assume so severe a type that the doctor will instruct the nurse to lower the patient's head so much as to make it the lowest point of the body, this position helping mechanically in the expulsion of irritating mucus. The nurse should not, however, place her patient in this position without permission from the doctor.

Teaspoons of lemon juice and glycerine often assist in clearing up the throat, and the nurse should bear in mind that hot drinks help to rid the throat of hard, "tight mucus."

In cases where the patient seems too exhausted to successfully cough up the collection of mucus in his throat, which gives rise to a feeling of impending suffocation, the nurse may have to apply a light, hot, tracheal poultice. In exhausted subjects, in the last stages of phthisis, or in the case of children who have not properly learnt how to expectorate, or whose bronchial tubes seem almost blocked by mucus, these tracheal poultices, changed every fifteen minutes, prove a great relief.

The over-burdened lung could not bear the weight of a chest poultice—placed over the trachea there is no sense of oppression, and great good may result. Gas should never be burned in the room of a lung patient, since this form of light has a quality of increasing mucous irritation and of aggravating cough. Gas fires, also, unless the chimneys are much better arranged than is usually the case, should not be used in bronchial or lung troubles.

In hot, dry, and irritable conditions of the mucous membrane a steam-kettle gives great relief, an added advantage of the kettle lying in the warmth the steam imparts to the air.

The local applications used in lung diseases vary so much under different medical hands that the subject is too wide to discuss here. It may be mentioned, however, that linseed and camphor poultices are much used in cases where it is necessary to stimulate and promote expectoration.

Powdered camphor is used in the proportion of 1 oz. of this to 1 lb. of crushed linseed. Where specially light poultices are necessary, bread crumbs may be employed in these camphor poultices, in the proportion of 1 oz. of camphor, ½ lb. of stale bread crumbs, and ½ lb. of crushed linseed. After the discontinuance of poultices, square sanitary towels may take their place. These are warm, light, and comfortable, and do not produce the "fluffy bed" which is apt to result from ordinary cotton-wool applications. These towels, too, serve admirably for application after the use of turpentine liniment or mustard leaves, and, covered with oil-silk, they make nice lung-jackets. For covering hot-water bags or bottles, and fulfilling multiple uses, these towels are very practical adjuncts to the sick room. In the tuberculous condition of the intestines, which sometimes attends cases of phthisis, sanitary square towels sewn together form excellent abdominal binders. Many doctors, however, prefer pine wool sewn in flannel for use in these tuberculous bowel cases.

Liniments and inhalations are so varied and numerous that no list of these can be given. A practical point for the nurse to remember, however, is that iodised oil suits delicate skins far better than does iodine liniment, and the oil stains linen much less than does the liniment.

The average nurse is expected to know how to prepare the pine wool respirator, used so much for phthisical patients. It may be as well, therefore, to remind nurses that these are made by taking two pieces of flannel, six to eight inches square, and quilting a layer of Lairitz pine wool between the flannel. Just before use, sprinkle with 10-15 drops of pine-oil, roll it up into the form of a tube, and let the patient breathe this for 7-8 minutes, several times daily.

The feeding of the bronchitic and phthisical patient is an important branch of knowledge to the medical nurse.

Many lung cases are attended with more or less gastric disturbance, and the nurse should always find out whether such dyspepsia and gastric trouble arises through the swallowing of sputum. This is a habit many lung patients contract, sometimes with very serious consequences. Children—especially those not old enough to have learned to expectorate—frequently get severe dyspepsia, vomiting, and diarrhœa through sputum swallowing. Special danger exists when the expectoration is fœtid or tuberculous; in the latter case, tubercle of the bowel may arise through swallowing the sputum. The first lesson a nurse needs to give to a child lung-patient is the important art of expectoration, which even quite small children easily learn.

Cases of bronchitis need specially good feeding—more especially in young and elderly cases. Old people not infrequently get paralysis of the bronchial tubes through exhaustion, and this possibility must, if any way possible, be warded off by good feeding.

Milk, cream, and fatty foods are indicated in lung troubles. *Café au lait*, to which whipped cream is added, brandy and whipped egg, clotted cream, cup custards with a liqueur, whisky and cream, Valentine and cream, are all excellent alike for the bronchitic and phthisical. Koumiss, milk, and malted foods, milk gruels, fresh cream, and whey and cream may all be used alternately.

Peptonisation may be necessary if dyspepsia be present. If cod-liver oil is ordered it should be given one hour after meals, with the addition of one-fourth of a liqueur, maraschino, noyeau, &c. When cod-liver oil causes nausea and indigestion, glycerine may be ordered as a substitute. Pancreatic emulsion, thoroughly mixed with warm milk and flavoured with coffee or a little liqueur brandy, or malt and cream are excellent substitutes for cod-liver oil.

Malted oatmeal (this is a food too little known and too little used in sick diet) made with milk, and invalid turtle soup, together with peptonised cocoa and milk, and the other foods mentioned, are indicative of the dietetic paths the nurse should lead the patient through.

### A Book and its Story.

### COCKNEY COLUMBUS.

In the "Cockney Columbus,"* Mr. Christie Murray records his impressions of the people and country "who have been the admiration of a lifetime." These impressions, scattered through a series of chapters, originally compiled as letters to friends at home, and written by him from America when travelling in search of health and recreation, are instinct with the freshness of first impressions. Many characteristic anecdotes are told of individuals, for whose pardonable patriotic shortsightedness everyone has some sympathy. An American is not far behind the average Englishman in a modest appreciation of the country of his birth. The glory of its institutions; the superiority of things British, or American, as the case may be, over those of any other country in the known world are facts of which there is no doubt. The following may serve to llustrate this from the American citizen's point of view:—

"A certain well-known mayor of New York, after a visit to Paris, returned home to announce to his fellow citizens that the paving of the streets of Paris was certainly inferior to that of New York," and here follows a word on the impression made on the stranger by this great city of the New World, which must surely be the noisiest in creation, in spite of its paving. We are told that the incessant banging of bells; rumbling and rushing of trains on the elevated railway, obscuring all street vista in its course overhead; the shrieking and roaring of engines over the stone pavements below; the rushing of traffic in every direction, form a pandemonium of sound which leads Mr. Murray to refer to our own busy Strand as "a garden of sleep in comparison." He arrives at the conclusion that this nation, nervous and highly strung as they are, must be singularly imperceptive to sound, and patient too, with an unusual patience, under circumstances which would drive the ordinary Britisher mad.

For instance, the overcrowding of the electric cars, "so thick along the line that they almost touch each other, rocking and pitching like boats at sea, ten people to every four seats, holding on by a miracle to the swaying straps for miles, while men crowd the outside platforms hanging on at the risk of their lives. These cars are the property of a business corporation whose rapacity in any other country would be checked in a week." Have we nothing analagous to this in the culpable overcrowding of the Underground trains at the opening and close of the business day in London? The true American revels in things on a colossal, magnificent scale—huge blocks of bricks and mortar, steamers of enormous dimensions, of which the *Priscilla*, plying between New York and Boston, is a very striking specimen: "The height of the Pyramids, more or less, not quite so long as from Hyde Park Corner to Charing Cross," with concrete-paved entrance-hall, wide staircases, a vast concert-hall capable of seating one thousand persons, twelve hundred beds—one almost expects to hear bed-rooms—furnished with palatial magnificence, and a huge restaurant, resembling a football field in dimensions. Constructed storey upon storey, it rises aloft till one wonders with the author by what secret power the huge unwieldy mass maintains its equilibrium. But it moves out in a stately dignified way, and keeps a record of from twenty to thirty miles an hour, in spite of its bulk and the unimportant detail of "its being built in defiance of all known laws of gravitation." (The simple soul wonders if the author has imbibed the American faculty of writing sarcastic.)

Everyone at home has an idea that in the luxury and comfort of railway travelling America is far ahead of us. Here is some of the author's experience (on the C.P.R.) which does not quite carry out the idea. He was disturbed at three a.m. to be asked if he would breakfast, and this, after a restless, uncomfortable night, in which the chief incidents were banging of bells (he wonders why Americans affect this noisy mode of signalling), and the periodical visitation of a dusky being throughout the dark hours, who, in his office of draught director to the cars, passed to and fro, admitting icy currents of air through the little hot chest in which he was reposing, stoically indifferent to the sufferings of the traveller. The result was that, "dumb and rebukit," he endured in silence an atmosphere "combining the austerities of a Greenland winter and the moist joys of a Cingalese summer." The undulations of the bed are likened to the surface of a mountain map. Wearied out, he fell into unrefreshing slumber; rose at half-past ten a.m., after a toilet performed under difficulties, and descended to breakfast. The buffet was closed until one p.m.! Later on, when opened, the choice of refreshments was limited to "hard-boiled eggs, tinned meats or sandwiches, dispensed with water and iced lemonade." Being the Sabbath stimulants were not retailed. One hardly wonders that the sorely-tried traveller in search of rest and recreation on turning in again found himself in a condition between freezing and melting, and "in a settled state of dyspepsia." Surely, the old simple barbaric modes, where nothing was expected, were better than these later autocratic developments in the art of luxurious, but distinctly uncomfortable, travelling. But the traveller forgets passing, if potent, discomforts in the delight experienced in the first view of the Rockies. "There are many and separate magnificences in the world of mountain landscape to rival most views which break upon you here, but for extent and continuance it would be hard to find an equal to the glory of this journey. I have never in my life experienced a fatigue of mind like that which fell upon me here." The description is a vivid and powerful one as it proceeds, and Mr. Murray is at his best under the spell of this marvellous region.

In Canada the author found himself more at home than in any other country he had visited. The houses, the flowers, the shrubs, the gardens were all English, with the same type of men and women that are found in Devonshire and Warwickshire. In the matter of electric light and kindred appliances they are ahead of us. Nor were they behind in the genial hospitality offered here, as elsewhere, to the modern Columbus.

America prides itself in being a free—in some cases an unusually free—country, and yet the autocracy in various departments under which it suffers is noticed as being something no ordinary European would rest content with for a moment. The autocracy of the "coloured gentleman" is an instance for which we in England can offer no parallel. It has even reached the high point, where, in the exercise of the honourable, if humble, calling of waiter, his demeanour is such, "that the idea of its being insolence vanishes, to such a state of perfection is it brought." And this is found also in the free and independent bearing of uncoloured "helps," and people of that class, by whom a respectful bearing is rejected as being synonymous with servility. The Press in America exercises a freedom unknown even in our country. An example of picturesque journalism, which would not obtain in high-class papers at home, is given. A lady's house takes fire; the reporter states, "Mrs. A—— is a large lady; in fact, adipose tissue is well represented on her comely person. She sleeps in the back parlour, and on Thursday night she had troubled dreams. She thought she was at the dentist's, had taken gas, and was suffocating. Just as she was breathing her last gasp, she awoke to find her door burst

* "Cockney Columbus." By D. Christie Murray. (Published by Downey and Co., York Street, Covent Garden. 6s.)

open, and all the male boarders rushing into the room. . . . It was a great rescue. They took her all at once, in one load; that is, it did not take two trips to carry her over." This is smart writing, before which ordinary journalism pales. What might have been a tragedy, becomes a comedy in the telling. But it seems hardly fair that the personal peculiarities of a portly lady should be made the subject of such ridicule.

Of course there is a tribute to the charm of the cultured vivid personality of the American woman, and the author accepts as admirable, Fanny Kemble's definition, that these "fragile, exotic-looking women, with their high, shrill voices, remind one of 'mice roaring,'" and confesses to having recourse to the simile to express what he frequently felt in regard to them. The American accent—or what is known to us as such—is not present in the Boston lady, who, with her "gentle, unobtrusive air, graceful, easy manner in conversation," suggests the English lady of a past generation.

The closing chapters of this entertaining book are devoted to the Antipodes. These appeared previously in the *Contemporary Review*, and raised some hostile criticism at the time, but many of the forecasts, in light of recent events, have been proved correct. The dominant note is a plea that the integrity of the colonies can only be maintained by federation; and the object considered most worthy of an Englishman's pursuit is the consolidation of the race, an opinion shared by many far-seeing men of the day. The book is well printed and got up, and one wherewith to beguile away a vacant hour pleasantly.

### The Zenana Bible and Medical Mission.

A BREAKFAST, with Lord Overtoun as president, was given in the Cannon Street Hotel on April 26th, at nine a.m., to welcome home Lord and Lady Kinnaird, Miss Morley, and the Rev. A. R. Cavalier. A large number were present, and after breakfast listened with much interest to the Chairman's bright survey of the growth of Indian missions. He spoke of the responsibilities of lifting up the natives, and remarked that if more money had been spent in evangelising India, forty millions of money would probably not have been needed to quell the mutiny. The difficulty of Englishwomen to realise the boons Christianity had conferred upon them vanished when the lot of India's women was compared with their own. There cows had more rights than women. They had a loveless marriage, a miserable age, an unregretted death, whilst the fate of a child widow was still more pitiable. Lord Overtoun said that he was often asked if the society had not enough workers already. Whereas, in fact, 13,000 more missionaries were still needed to send one to every 20,000 inhabitants. Lord Kinnaird followed with some reminiscences of his tour, the most noticeable point in his remarks being that relating to the cordial co-operation now existing between the missions and the civil authorities, a result gained by the high spiritual standard attained by all working there—lady doctors, nurses, and missionaries. The rise in the value of the rupee, the speaker explained, costs the mission £2,000 a-year more, and money is much needed in every department of the work. The hospitals are overflowing, and structural repairs and additions cost a good deal. The Rev. A. R. Cavalier then gave further particulars, and Mr. W. T. Paton made a humorous appeal for the completion of a sum of £5,000. Cheques and promises of subscriptions came freely in, and the meeting terminated with prayer and a benediction.

The annual report of the society will be read on Friday afternoon, April 29th, 1898, at three p.m., at the New Hall, 26, George Street, Hanover Square, W., to which any interested in the work are welcome. A missionary meeting, illustrated by limelight views, will be held in the same place on the same day at eight p.m. Admission free.

### American Women and War.

IT may be interesting, at the present time, to recall a few facts with reference to the noble part played by American women in the care of the wounded soldiers engaged in the Civil war. Now that the United States is at war with Spain, there is no question that they are in a position to make their nursing and hospital arrangements on a scale of perfection never before equalled in the wars of nations. So far back as 1861, the Americans were able to equip their field service and various agencies for giving First Aid to the wounded in battle in an admirable manner, which has served as a pattern to every other country. And the nursing service at that time, it must be remembered, was largely the work of amateurs. To-day the U.S.A. possesses a vast army of skilled and efficient nurses, who can and will be put into action at a few days' notice.

In the Civil War, to the women of New York belongs the honour of having taken the first steps towards the organisation of the vast nursing array which in North and South did such noble service on the field. It was the women who started the register of male and female nurses for work among the wounded, and it was through the energy of the women in holding vast bazaars for the benefit of the sick and wounded soldiers, that such huge sums were collected to supplement the Government hospital grants. Women provided Homes of Rest for soldiers on the march, and ladies who had never before faced the realities of life cheerfully worked for weeks in floating hospitals crowded with cases of typhus. They also set up asylums at the various railway stations, where soldiers passing through were provided with clothing and necessaries, and, if sick or convalescent, were carefully looked after, nursed back to strength, and provided with the money to enable them to rejoin their regiments.

These "Wayside Homes" proved to be of the utmost value both from the moral and physical points of view. In addition, waggon-kitchens were established along the routes of march, and here women of every class occupied themselves day and night in preparing and cooking food for the passing troops, thus providing nourishment to many who had neither the time nor the strength to prepare it for themselves.

Bands of women, too, devoted themselves to procuring and transporting constant supplies of fruit and fresh vegetables for the use of the armies, and it was largely owing to their valuable assistance that not a single soldier died of scurvy.

Women whose age, condition, and want of skill prevented them from joining the nursing ranks or visiting in the hospitals busied themselves with preparing packets of envelopes containing paper and pencils for the use of the sick who wished to communicate with anxious friends.

Others went the rounds of the hospitals, and relieved the over-taxed nurses by taking on themselves the duty of writing and answering the letters of those who were too ill to do this for themselves, and it was to such noble, thoughtful women that last dying messages, trinkets, and souvenirs were entrusted for the consolation of the friends of the dead soldier.

Of the devotion of nurses and amateurs who went through a hurried training of a few weeks in order to qualify for posts in the military hospitals it is impossible to speak, for whole volumes might be written of the heroism and self-sacrifice displayed by the women of the Civil War. With such examples before us of what has been accomplished by American women in time of war, it is quite safe to predict that now that an outbreak of war has occurred between the United States and Spain, that the trained nurses of the former country will show themselves second to none in bravery and devotion to duty.

### Nurses for Board Schools

The Queen's nurses, who visit the poor in their own homes, are known and respected throughout the length and breadth of the land; but there is one Queen's nurse who has struck out a new branch of work, and in so doing given an example which should be widely followed. From the Central Home in Bloomsbury, every school day there starts a nurse to visit some of the poorest schools in the neighbourhood. She has at present six on her list—Vere Street, Great Wild Street, The Pulteney, Hart Street, Crown Court, and Tower Street —and each school is visited two or three times a week.

It was the privilege of the writer to accompany the nurse on her rounds on March 15th last, and the first Board school visited was the cramped and dingy old building in Vere Street, Clare Market. We made our way to the teachers' room of the boys' department first, and nurse spread out a newspaper on the table, and laid thereon her few appliances. A tin biscuit-box is kept at the school, from which cotton wool, strapping, &c., were produced, and the nurse carried round with her in her bag boracic, carbolic, nitric acid, &c., in bottles.

The boys came in four at a time, and had their broken knees and sore heels dressed or their inflamed eyes washed or their ears syringed. The cases were all slight (except one of inflamed eyes), and the nurse was inclined to be apologetic about them. But quite needlessly, for the head master came in to bear witness to the regular attendance which the nurse's visits did so much to maintain.

An amusing incident was nurse's confession that before she took up school work she had regarded the teachers with dread, and ever expected to see them cane in hand. The splendid humanity of the staff at the Vere Street school had quite dispelled this illusion. A kettle of hot water, a basin, and a cup are all that the school need supply to help the nurse in her work, but of course, without the co-operation of the teachers, she can do nothing. But that co-operation has never failed her; the teachers are only too glad that the unfortunate little children under their charge should have the benefit of the nurse's kind ministrations.

From the boys' department we went down to the girls', and then to the infants' department. In all 26 children were seen in 1½ hours, the majority of the cases running on these lines:—

(1) A. B. Bathe eyes with boracic lotion, put boracic ointment on lids, improvise shade, ask not to be allowed to read or write.
(2) C. D. Sore knee washed with carbolic, piece of lint with zinc ointment put on with bit of strapping. Warts on finger touched with nitric acid.
(3) E. F. Inflamed Finger.—Wash boracic fomentation, oil silk, bandage. Discharging ear syringed.

Not long ago the London School Board started a day school for truants, in Drury Lane, and has now been making enquiries why they can get no truants from the neighbouring streets to fill it. The answer is given at Vere Street, where the attendance has gone up from 67 to 95 per cent., owing to the favour in which the school is held, because of the humane methods of the teachers there, and their kindly interest in their little scholars.

The next school visited was Great Wild Street, where 27 children were attended to, the cases, on the whole, being very slight.

Next to Crown Court, where 18 children were seen, making a total of 71 cases attended to between nine and twelve—the morning hours for school. The nurse noted certain cases of sore heads, &c., which she would have to follow up at home, and also went off to visit a few scholars who were too ill to attend school. Now, granted that much of the work done looks trivial, it is obvious that all this trivial work is largely preventive, and that the best way of making the people healthy is to catch small ailments at the beginning. The sore heel soon becomes poisoned if left exposed to the dirt of the streets; the inflamed eyes, alas! often lose all power of seeing simply through neglect. The nurse can tell at a glance whether a child should be sent to a doctor or not, and she can recognise the early symptoms of those infectious diseases which so often devastate our schools. In the matter of cleanliness, the women teachers especially bear grateful testimony to the benefits resulting from the teaching and influence of the nurse.

There is a scheme on paper for starting a "School Nurses' Society" to supply visiting nurses to all the elementary schools in very poor districts where the teachers feel the need of a nurse's services. Possibly this society might affiliate with the Q.V.J.N.I., and supply the funds while the Queen's nurses did the work. Certainly the work is worth the doing, to judge by a morning's experience of what one nurse can do in this way.

### Appointments.

### MATRONS.

General Hospital, Birmingham.—Miss Mary Esther Jones was appointed Matron of the above hospital on April 22nd, having been selected out of sixty candidates. She was trained at the Royal Southern Hospital, Liverpool, 1883-6, and was subsequently sister at Cardiff Infirmary, and sister and night-superintendent Monsall Hospital, Manchester. Miss Jones has since then held the following appointments: Matron, Devizes Cottage Hospital; matron, the Middlesbro' Sanatorium; matron of the Metropolitan Asylums Board Eastern Hospital, Homerton; and matron of the Park Hospital, Hither Green, under the same Board.

South Lincolnshire Hospital.—On the 21st inst. Miss Constance Bobby was appointed Matron of the above hospital. She was trained at Addenbrooke's Hospital, Cambridge, and took a further course of special training for three months at the Victoria Hospital for Children, Chelsea. From 1892 to 1894 she took charge of the children's wards at Addenbrooke's Hospital. From that date to 1895 she was charge-nurse at Chalmer's Hospital, Edinburgh, when she was appointed matron of the Cottage Hospital, Nuneaton.

### Minor Appointments

Infectious Hospital, Grenoside, near Sheffield.—Miss Mary Duffus has been appointed temporary Matron of this hospital. She was trained at St. Bartholomew's, and has recently been matron at St. Mary's Infirmary, Holloway.

### Presentations.

After six years' work at the Broomhill Home for Incurables, Miss Ewing was presented with several handsome gifts upon her retirement from the post of Matron. One morning the patients gave her a handsome travelling bag, fitted with brushes, and a combined purse and card-case, each bearing her initials. In the evening, after tea in the nurses' sitting-room, the household staff gave her, together with a most complimentary letter of regret, a fully-fitted dressing-case, also inscribed with her initials; and at a subsequent meeting of the Ladies' Committee Miss Ewing was the recipient of a table and spinning chair, beautifully carved.

### Wants and Workers.

"The Parish Nurse, Slough," asks if anyone will give an invalid boy of eight a spinal carriage to enable him to get into the fresh air.

### For Reading to the Sick.

"LET patience have her perfect work that ye may be perfect and entire, wanting nothing."—*James* i. 4.

**Verses.**

Why restless, why so weary,
My soul, why so cast down?
Is all around so dreary,
And hath the Cross no Crown?
Where is the God who found thee,
Who once could make thee glad?
Are not His Arms around thee,
And wherefore art thou sad?

—*Clewer Manual.*

Grant Thou this patience, Jesus to me!
Grant Thou Thy graces, my safeguard to be!
So that in all things Thy will may be mine,
Bearing all troubles because they are Thine.

—*J. M. Neale.*

God doth not bid thee wait
To disappoint at last;
A golden promise, fair and great,
In precept-mould is cast.
Soon shall the morning gild
The dark horizon rim;
Thy heart's desire shall be fulfilled,
Wait patiently for Him.

—*F. R. Havergal.*

O comrade bold of toil and pain!
Thy trial how severe;
When sever'd first by prisoner's chain,
From thy loved labour sphere!
Say, did impatience first impel
Thy heaven-sent bond to break?
Or couldst thou bear its hindrance well,
Loitering for Jesu's sake?
Oh might we know! for sore we feel
The languor of delay,
When sickness lets our fainter zeal,
Or foes block up the way.
Lord! who Thy thousand years dost wait,
To work Thy thousandth part
Of Thy vast plan, for us create
With zeal, a patient heart! —*Newman.*

Let us be patient! These severe afflictions
Not from the ground arise;
But oftentimes celestial benedictions
Assume this dark disguise. —*Longfellow.*

**Reading.**

Patience is the endurance of any evil, out of the love of God, as the will of God. The offices of patience are as varied as the ills of life. We have need of it with ourselves and with others; with those above and below us, and with equals; with those who love us and those who love us not; for the greatest things and the least; against sudden trouble and under daily burdens; disappointment as to weather or the breaking of the heart; in weariness of body, in wearing of soul; in our own failure and others failure to us. In all these things, from childhood's little troubles to the martyr's sufferings, patience is the Grace of God, whereby we endure evil for love of Him, and keep still and motionless not to offend Him.—*Dr. Pusey.*

Patience makes the soul to be of one mind with God, and sweetens all the ills of life. It casts the light of Heaven upon them, and transforms them into good. It made the bitter waters sweet, the barren and dry land fruitful. Desolation it makes loneliness with God; the parching of sickness to be the fire of His love; weakness to be His strength; wounds to be health; emptiness of all things to have all things from Him; poverty to be true riches; His deserved punishments to be His rainbow of mercy; death to be His life.—*Dr. Pusey*

### Notes and Queries.

**The contents of the Editor's Letter-box have now reached such unwieldy proportions that it has become necessary to establish a hard and fast rule regarding Answers to Correspondents. In future, all questions requiring replies will continue to be answered in this column without any fee. If an answer is required by letter, a fee of half-a-crown must be enclosed with the note containing the enquiry. We are always pleased to help our numerous correspondents to the fullest extent, and we can trust them to sympathise in the overwhelming amount of writing which makes the new rules a necessity. Every communication must be accompanied by the writer's name and address, otherwise it will receive no attention.**

*Training School in Melbourne.*

(40) Can you give us the name of a training school in Melbourne or any other healthy part of Australia? Do you consider we could do better at private nursing out there than in England? Will you also give us the name of whom to address *re* being trained for Red Cross nursing?—*Two Orphans.*

The Melbourne General Hospital. We doubt, however, if the matron would engage probationers without an interview, and it would be most unwise to go out without a definite engagement. Private nurses there stand no better chance than in England. The Red Cross nurses are engaged after training.

*School of Massage.*

(41) Would you kindly let me know if there is a recognised school of massage in London?—*Nurse Brown.*

No. Write to the Society of Masseuses, 12, Buckingham Street, Strand.

*Probationers Under 21 Years of Age.*

(42) I am 18½ years of age, and wish to become a nurse. Can you tell me of any hospital or infirmary that take probationers at my age?—*Seaside.*

Your age would preclude you from being accepted at any but a children's hospital. We should advise you to write to the Matron of the Alexandra Hospital for Children with Hip Disease, Queen Square, London, W.C., where they take young probationers.

*The Norland Institute.*

(43) Could you give me a little information about the Norland Institute?—*Nurse Rose.*

If you write to Miss Sharman, Principal, the Norland Institute for Training Nursery Nurses, 29, Holland Park Avenue, London, W., you will be able to obtain full particulars.

*Who is Eligible for the Hospital Convalescent Fund?*

(44) 1. Is a nurse 43 years of age eligible for the Hospital Convalescent Fund? She is suffering from asthma, and is anxious to resume work as soon as possible. 2. Is there any hospital where nervous diseases are specially dealt with?—*Nurse B.*

1. Every trained nurse who requires help after illness to enable her to return to work is eligible for assistance from the Hospital Convalescent Fund. She must apply to the Secretary, at THE HOSPITAL Office, enclosing a certificate from her medical attendant, and two references from householders; and she may state what climate is recommended as best suited to her case. If these requirements are satisfactorily complied with, the applicant is usually sent to a convalescent home for three weeks, and a small sum is granted towards expenses. 2. There are several hospitals for nerve diseases. The National Hospital for the Paralysed and Epileptic, Queen's Square, Bloomsbury, is the most important.

*Proper Doses of Commonly Used Drugs.*

(45) Can you recommend me a book to direct me in administering the right dose of simple medicines, such as Gregory's mixture, senna, &c.? I have charge of a large home of healthy children, and a medicine chest, but with no directions as to the use.—*Daisy.*

"Materia Medica and Therapeutics," by Mitchell Bruce, would give you full information; much more than you want. It could be procured from the Scientific Press, 28 & 29, Southampton Street, Strand. You might also write and ask the druggist who supplies the medicines for labels to affix to the bottles. He would possibly supply them free of charge. But healthy children do not require physic.

*Probationer at 37.*

(46) Is a woman 37 years of age too old to become a probationer? I am experienced in domestic nursing, and have been head nurse of three to children. Will any hospital accept me?—*Clara.*

Hospitals, as a rule, decline probationers over 30-35, because it is extremely rare to find one who succeeds well after the first youth is passed. Addenbrooke's Hospital, Cambridge, is one, however, Chichester Infirmary another, and Cumberland Infirmary a third; so that the door of the nursing profession is not shut to "Clara" yet if she has set her heart upon entering it. But she must remember that she will be 40 before she is trained, and she can now command almost as good wages as a children's nurse as she would then as a trained nurse.

*To Become a Queen's Nurse.*

(47) Please inform me where I can obtain further training with a view to becoming a Queen's Nurse.—*Stanley.*

Write direct to Miss Peter, Queen Victoria's Jubilee Institute for Nurses, 1, St. Katharine's Royal Hospital, Regent's Park, N.W.

*Homes for Incurables, Paris.*

(48) Can you tell me if there are any homes for incurables in Paris where a French sailor (destitute) suffering from paralysis after an accident could be received, and where he could be taught a trade? (2) Is Condy's fluid powerful enough to disinfect drains?—*Nurse Keen.*

(1) We will make inquiries. (2) If used in sufficient strength. Carbolic acid (Calvert's No. 5) is generally used for this purpose.

*Compensation on Death.*

(49) Will L. Phillips kindly repeat her question more clearly? We do not understand whose death is referred to—the employer's or the nurse's.

